# The intersection of disability status and rurality in American Indian/Alaskan Native communities

**DOI:** 10.3389/fresc.2022.875979

**Published:** 2022-08-02

**Authors:** Genna M. Mashinchi, Emily C. Hicks, Arin J. Leopold, Lillie Greiman, Catherine Ipsen

**Affiliations:** University of Montana, The Rural Institute for Inclusive Communities, Missoula, MT, United States

**Keywords:** disability, American Indian/Alaskan Native, rural, health disparities, health equity, social determinants of health

## Abstract

There is a noteworthy gap in the literature regarding disability in rural American Indian/Alaskan Native (AI/AN) communities. This is significant, as many tribal lands are in rural areas and AI/AN individuals experience some of the highest prevalence rates of disability. To address this gap, we used descriptive statistics to examine the intersection of AI/AN and rurality in disability prevalence. Results indicate that rural counties have the highest prevalence of disability for both Whites and AI/ANs and that AI/ANs experience higher prevalence rates than Whites. However, further analysis indicates that county makeup (counties with high prevalence of AI/AN in the general population) moderated this relationship. Specifically, rural counties with populations of at least 5% AI/AN had lower prevalence of AI/AN disability compared to counties with populations with less than 5% AI/AN. Further analysis is needed to unpack this relationship, but results might suggest that AI/AN communities may feature resilient and protective attributes, moderating the amount of disability experienced in rural AI/AN communities.

## Introduction

Disabled people^1^ have historically been stigmatized and marginalized throughout the United States (1). While important legislation such at the Americans with Disabilities Act, the Rehabilitation Act, and the Olmstead Decision have led to significant progress in increasing disability awareness and promoting full inclusion into American society (2), inequities in access and inclusion persist (1, 3). Marginalization is compounded for minoritized groups (e.g., American Indians/Alaskan Natives, Blacks, Hispanics) who also experience disability (4). In fact, data from four national population surveys conducted in 2015 revealed that minoritized individuals—such as individuals of an ethnic or racial minority—report disability at disproportionately higher rates than White individuals (4).

Despite increased disability awareness and literature regarding disparities faced by individuals with disabilities, there is a significant knowledge gap regarding disability among American Indian/Alaskan Natives (AI/ANs), particularly those living in rural areas. U.S. Census data indicate that 16% of AI/ANs report disability across counties, and that prevalence rates increase as counties become more rural (5). This finding aligns with descriptive statistics that show individuals in rural counties have higher prevalence of disability across racial groups, and that the most rural (non-core) counties have higher prevalence of disability, compared to less rural (micropolitan) and urban (metropolitan) counties (5). While the majority (63.5%) of AI/AN individuals reside in urban areas (6), the remaining rural population represents nearly one million (982,517) people with 17% (nearly 170,000) reporting disability (6).

The limited research looking at the intersection of AI/AN identity, disability, and rurality has focused on health outcomes, rather than environmental factors (7). This is problematic because disability is shaped by the interaction between functional limitation (e.g., difficulty walking, grasping, or concentrating) and environmental factors (e.g., community characteristics, access to resources, social stigmatization, and exclusionary policies). While symptoms of specific health conditions can result in a variety of functional limitations, it is the interaction of those limitations with the surrounding environment that produces the incidence and severity of disability (6, 7).

Community characteristics (e.g., sociodemographic and access factors) that rural AI/AN individuals encounter are critical to understanding the disability experience (8), and shape the need for studies examining disability disparity from a community-specific perspective. Of note, Henning-Smith et al. (9) examined premature death rates and the intersection of rurality and race in AI/AN, White, and Black communities from a county-level perspective. The authors found that rural counties with a majority of AI/AN residents experienced significantly higher premature death rates, even after adjusting for community-level covariates. However, disability rates were not examined.

We explored the prevalence of disability for the AI/AN population living in rural communities. Given past findings that AI/AN populations report higher levels of disability and disproportionately live in more rural areas—which also have higher prevalence of disability (5)—we hypothesized that there would be a positive association between AI/AN prevalence and AI/AN disability prevalence rates across geographic locations.

## Methods

### Sample

The study sample consisted of 3,220 counties across the United States and the unit of analysis was at the county vs. individual level.

### Procedure

All data was from the American Community Survey 5-year estimates (2015–2019) (6). The American Community Survey is a cross sectional survey operated by the US Census Bureau. It uses an annual rolling sample, collecting data on 2.5% of the US population per year for an aggregated sample of 12.5% of the US population in the 5-year estimates. A 2015 report found that while there is increased room for estimate error for small geographies, the coefficients of variation for AI/AN communities were similar to others of similar sizes and deemed reliable (10).

Race/ethnicity was defined as the percentage of the county population of each racial category based on ACS data reports. We used AI/AN and White racial categories for these analyses.

We accessed 2018 cartographic boundary shapefiles for geographic analyses (counties, states, and tribal areas) from the US Census Bureau's geography downloads.

We created an AI/AN county makeup variable to analyze AI/AN populations more closely. This was a binary coded variable, such that counties with ≥5% of residents identifying as AI/AN were classified as having “high AI/AN” populations, and the remaining counties were classified as “remaining.”

Disability was defined using the American Community Survey six question set asking about functional ability and supports. If a response was yes to at least one of the following six American Community Survey questions, we classified individuals as having a disability:

1. Are you deaf, or do you have serious difficulty hearing?
2. Are you blind or do you have serious difficulty seeing even when wearing glasses?
3. Because of a physical, mental, or emotional problem, do you have difficulty remembering, concentrating or making decisions?
4. Do you have serious difficulty walking or climbing stairs?
5. Because of a physical, mental, or emotional problem, do you have difficulty dressing or bathing?
6. Because of a physical, mental, or emotional problem, do you have difficulty running errands alone, such as visiting a doctor's office or shopping?

We classified counties as metropolitan, micropolitan, and non-core using the United States Office of Management and Budget (OMB) classification. OMB classifies counties as metropolitan and nonmetropolitan based on population data collected by the U.S. Census Bureau. The OMB defines metropolitan counties as counties with an urban core of over 50,000 people. Metropolitan counties are generally considered to be urban. Non-metropolitan counties are classified into two rural subclassifications: micropolitan counties, with urban populations between 10,000 and 50,000 people, and non-core counties as all remaining counties with urban cores <10,000 (11).

### Data analysis

Analyses were conducted in ArcMap, Version 8.1 and IBM SPSS Statistics for Windows, Version 28.0. We used ArcMap to visually examine county-level geographic distribution of AI/AN disability prevalence. We used SPSS to run descriptive statistics, *t*-tests, and Pearson *r* correlations to explore the relationship between disability and rurality in AI/AN populations.

## Results

**Figure 1:**
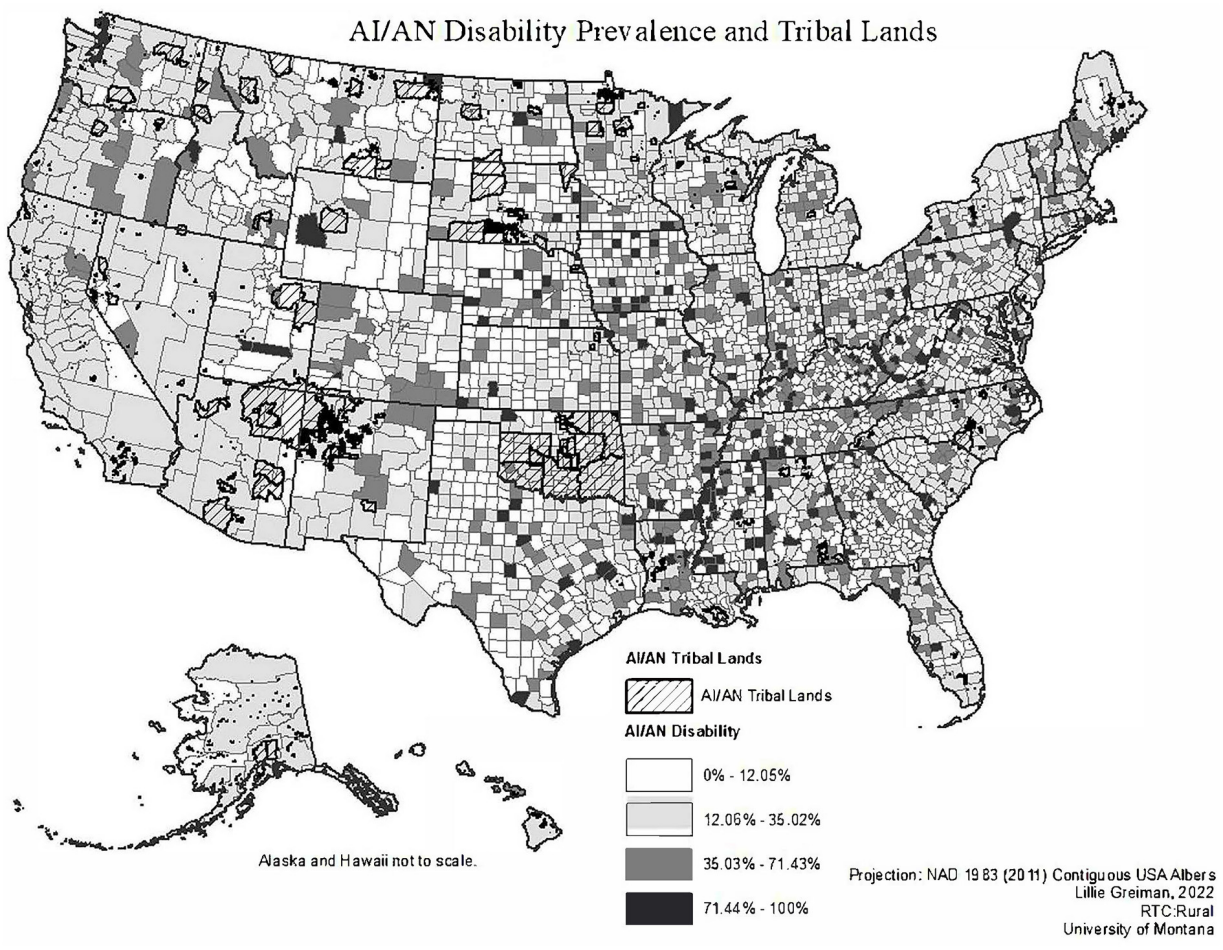
Map of AI/AN disability prevalence overlayed with AI/AN tribal reservation and trust lands.

Figure 1 illustrates the geographic variation in AI/AN disability prevalence with an overlay of Tribal Trust and Reservation lands. There does not appear to be any visual correlation or relationship between higher rates of AI/AN disability and counties overlapping tribal lands where a significant proportion of AI/ANs reside.

A Pearson *r* correlation analysis of counties revealed a significant negative correlation between the AI/AN concentration in the county population and the AI/AN county disability prevalence, *r*(3,218) = −0.061, *p* < 0.001. This finding indicates that higher concentrations of AI/AN in the county population were associated with lower rates of reported disability among AI/AN county residents.

**Figure 2:**
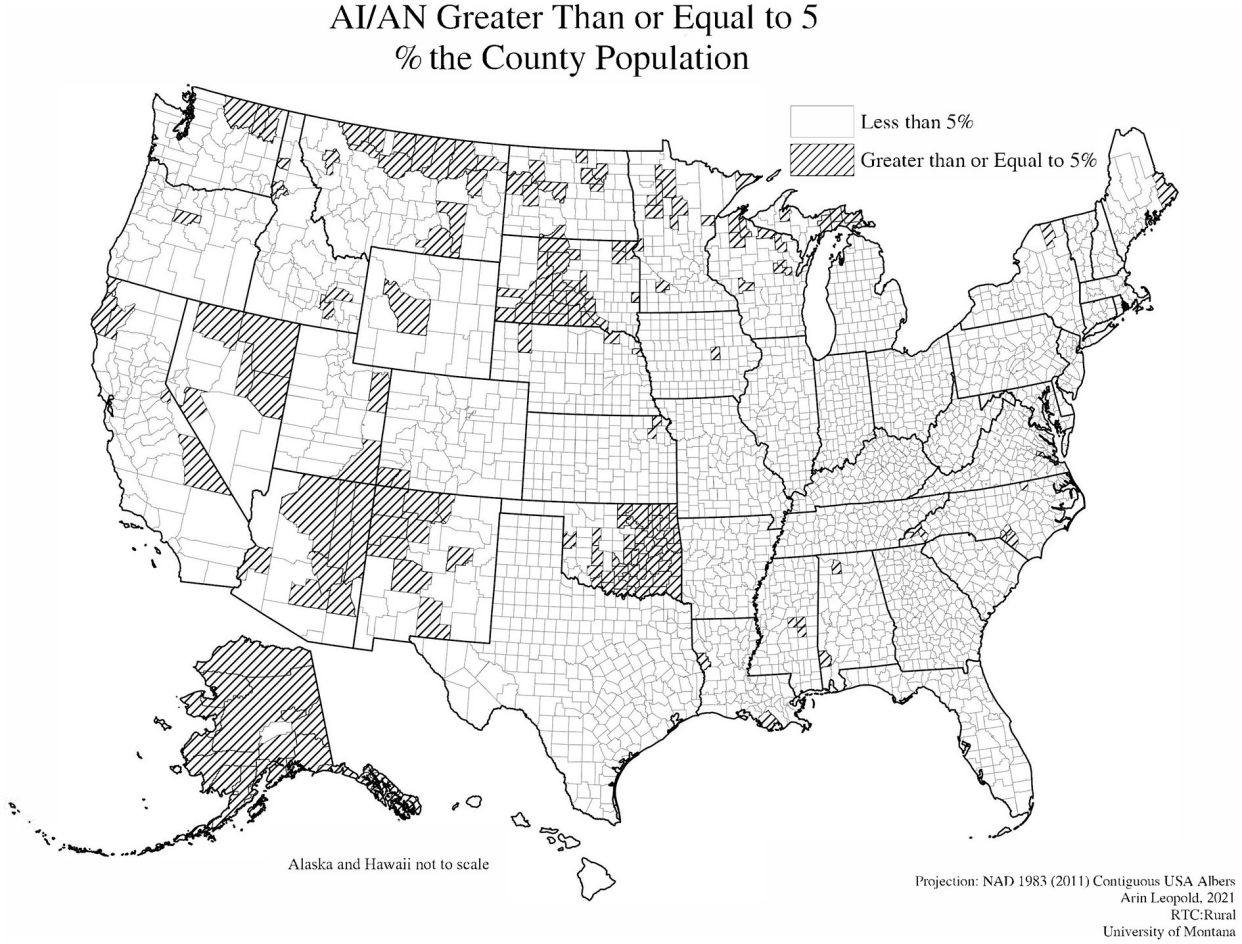
Map of high AI/AN counties.

Figure 2 shows a visual map representation of counties with high AI/AN populations. There were 211 counties with AI/AN populations of 5% or more. These counties are located primarily across the western United States with high concentrations in Alaska, Oklahoma, South Dakota, Montana, Arizona, and New Mexico.

Table 1 compares disability rates between “high” and “remaining” counties using group comparison *t*-tests based on prevalence of AI/ANs in the general county population. Results indicate that the disability prevalence for AI/ANs in metropolitan, micropolitan, and non-core counties with “high” prevalence of AI/AN populations (5% or more) had significantly lower prevalence of AI/AN disability relative to “remaining” counties. In fact, the prevalence of AI/AN disability in high AI/AN counties is equal to or lower than prevalence of White disability prevalence across metropolitan, micropolitan, and non-core county groups. In contrast, Whites in metropolitan counties with “high” prevalence of AI/AN populations had significantly higher prevalence of disability relative to “remaining” counties. Differences were not statistically significant for differences in micropolitan and non-core counties.

**Table 1:** *T*-test comparisons of disability prevalence among AI/ANs and Whites in “high” vs. “remaining” AI/AN counties^*^.

	**“High”** **AI/AN counties** ***M* [*SD*]**	**“Remaining”** **AI/AN counties** ***M* [*SD*]**	***T***	***p***	**All counties ^†^** ***M* [*SD*]**
**AI/AN disability prevalence**					
Metropolitan	16.4 [3.5]	21.0 [19.2]	5.31	≤ 0.001	20.9 [19.0]
Micropolitan	16.4 [4.5]	24.4 [24.0]	6.80	≤ 0.001	23.9 [23.3]
Non-Core	16.4 [6.9]	24.4 [29.8]	7.61	≤ 0.001	23.6 [28.4]
					
**White disability prevalence**					
Metropolitan	16.8 [3.6]	14.8 [4.3]	2.98	0.006	14.9 [4.3]
Micropolitan	17.4 [4.6]	16.2 [3.9]	1.59	0.118	16.3 [4.0]
Non-Core	16.9 [5.7]	17.7 [4.7]	1.68	0.095	17.6 [4.8]

* *“High” counties are characterized as having general populations with at least 5% AI/ANs, whereas “remaining” counties have populations with less than 5% AI/ANs.*

† *We used one-way ANOVA and post-hoc analyses to examine disability prevalence for AI/ANs and Whites across metropolitan, micropolitan, and non-core counties. Disability prevalence was significantly higher for AI/ANs living in non-core relative to metropolitan counties (p = 0.005) and micropolitan relative to metropolitan (p = 0.008), but not for noncore compared to micropolitan counties (p = 0.744). Disability prevalence was significantly higher for Whites living in noncore relative to micropolitan (p ≤ 0.001) and noncore relative to metropolitan (p ≤ 0.001), and micropolitan relative to metropolitan (p ≤ 0.001)*.

Table 1 also shows comparison across all counties (without grouping them based on concentration of AI/ANs). We used one-way ANOVA and *post-hoc* analyses to examine disability prevalence for AI/ANs and Whites across metropolitan, micropolitan, and non-core counties. Disability prevalence was significantly higher for AI/ANs living in non-core relative to metropolitan counties (*p* = 0.005) and micropolitan relative to metropolitan (*p* = 0.008), but not for non-core compared to micropolitan counties (*p* = 0.744). Disability prevalence was significantly higher for Whites living in non-core relative to micropolitan (*p* ≤ 0.001) and non-core relative to metropolitan (*p* ≤ 0.001), and micropolitan relative to metropolitan *(p* ≤ 0.001). We also explored the interaction between county classification and proportions of AI/AN population. We found a statistically significant interaction between non-core and high AI/AN (*p* = 0.003). This suggests that although non-core counties have higher rates of disability, the interaction of non-core and high AI/AN significantly lowers the disability rate.

**Figure 3:**
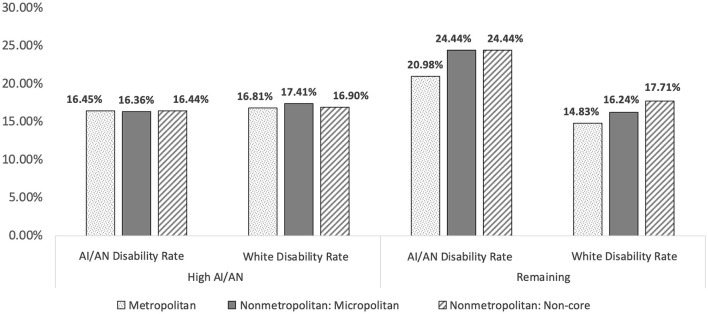
Comparisons of county makeup, county classification, and AI/AN disability rates.

Figure 3 further illustrates Table 1 results in a bar chart format.

## Discussion

When examining counties all together, there were notable differences between the prevalence of disability in AI/AN and White racial groups, as AI/ANs reported disability prevalence rates of 20.9, 23.9 and 23.6% and Whites reported rates or 14.9, 16.2, and 17.6% across metropolitan, micropolitan and non-core counties. These data illustrate a common finding that disability rates are high among rural people and for AI/ANs relative to Whites.

However, closer examination of the data (see Table 1; Figure 3) are contrary to our proposed hypothesis and past research (5). Specifically, a different pattern of disability prevalence emerged in counties with higher percentages of AI/ANs in the general population. Across metropolitan, micropolitan, and non-core counties, the disability prevalence in AI/ANs was significantly lower in counties with “high” AI/AN populations (≥5%) relative to counties with lower AI/AN populations (<5%). Additionally, reported disability prevalence rates in “high” AI/AN counties where actually lower for AI/ANs relative to Whites.

This finding may be due to two related hypotheses in the literature: (a) the belongingness hypothesis, which states that strong connections with others have strong effects on individuals, both emotionally and cognitively (12); and (b) cultural protective factors, in which the belonging aspect of being part of a culture and its traditions results in specific protective factors for those that belong, such as emotional wellbeing and resiliency in the face of negative outcomes (13–15). Previous research has found that a sense of belonging mitigates against negative life satisfaction that results from disability-related discrimination (16). In AI/AN communities, cultural traditions are reported to be protective factors because they provide a sense of purpose, a support system, comfort, companionship, and belongingness (17), which may lead to a reduced experiences of environmental barriers for those with health conditions, creating lower prevalence of disability. It is possible that the vehicle through which belongingness contributes to reduced disability prevalence is through mental health. Unfortunately, we were unable to examine the difference between race within specific disability type (e.g., mental illness, mobility difficulties) because we were working with county level prevalence data rather than individual-level data.

Further, the finding that AI/AN individuals living in high AI/AN counties experienced lower prevalence of disability, compared to White individuals, might illustrate that an intersectional protective factor may exist. Again, it should be noted that disability and health are not interchangeable terms. Disability is the interaction of environmental factors (i.e., inaccessible buildings, lack of public transport, exclusionary policies, and practices) and a functional limitation brought on by a health condition which results in disability (18). It is plausible that high AI/AN communities are more attentive to the environmental factors that impact community members with disability, thus creating fewer environmental barriers, and leading to lower prevalence of disability. Given that we did not use inferential statistics or an experimental design, the authors were unable to speak to causation or confounding variables, such as differences in federal- and state-level policies or differences between federal- and state-recognized tribal lands. Future research studies examining a possible protective factor should include these variables to fully examine this relationship.

Findings also suggest the importance of economic resource considerations. Rural counties generally experience sparse economic resources and opportunities (19). This leads to broad, community-wide health disparities due to a lack of funds for insurance, food, and other factors that impact health (3). This is in line with recent data from the U.S. Census Bureau that indicates that while generally, rural Americans experience lower household incomes than urban households, those living in rural areas have lower poverty prevalence than individuals in urban areas (20). Taken together, this suggests a protective factor of living in rural communities despite fewer economic resources. Protective factors may include a greater sense of community connectedness, support, and a lower cost of living in rural areas (21).

### Limitations and future research

The present study includes limitations for consideration when interpreting results. First, the study used county level rates which limit the ability to draw conclusions based on individual-level factors. Second, there were few AI/AN individuals represented in several counties, which introduced higher margins of error for interpreting results. However, our findings do have important implications for public health policy. To reduce disability disparity in areas with lower prevalence of AI/AN individuals, economic resources and community factors must be considered alongside individual considerations in future research. Additionally, as disability prevalence rates were higher for rural areas compared to metropolitan and micropolitan areas, disbursement of resources to address environmental factors in rural communities must be considered.

Further, data that does exist is often from an individual and deficit-based perspective, which tends to place blame on AI/AN individuals for health disparities, rather than considering community contextual factors. Data equity should be encouraged by increasing community-focused, asset-based, and culturally responsive data gathering in AI/AN communities. Similarly, additional research is needed to understand the intersectional protective factors that exist for AI/AN individuals residing in counties with high AI/AN populations. Finally, our study could not explore the influence of culturally-specific and culturally safe health care provisions on the prevalence of disability. Future research should aim to examine this in the context of disparities for AI/AN individuals.

## Data availability statement

This study used publicly available data. The metadata or the description of how we created variables will be included in ICPSR and will be available upon request.

## Ethics statement

We used secondary data analyses from U.S. Census data and was thus exempt from review by the Institutional Review Board.

## Author contributions

Data aggregation, data analysis and cleaning for the project was done by LG. The idea for the project was generated by LG and GMM. The writing was done by GMM, ECH, and LG. The tables and figures were done by AJL, LG, GMM, and CI. Critical review of the paper, suggested edits, and review and final edits were done by GMM, ECH, LG, AJL, and CI. All authors contributed to the article and approved the submitted version.

## Funding

This work was supported by the Research and Training Center on Disability in Rural Communities (RTC:Rural) under a grant from the National Institute on Disability, Independent Living, and Rehabilitation Research (grant number 90RTCP0002-01-00). NIDILRR is a Center within the Administration for Community Living (ACL), Department of Health and Human Services (HHS). The research does not necessarily represent the policy of NIDILRR, ACL, or HHS and one should not assume endorsement by the federal government. ECH receives/received support from Montana INBRE – an Institutional Development Award from the National Institute of General Medical Sciences of the National Institutes of Health under Award Number P20GM103474.

## Conflict of interest

The authors declare that the research was conducted in the absence of any commercial or financial relationships that could be construed as a potential conflict of interest.

## Publisher's note

All claims expressed in this article are solely those of the authors and do not necessarily represent those of their affiliated organizations, or those of the publisher, the editors and the reviewers. Any product that may be evaluated in this article, or claim that may be made by its manufacturer, is not guaranteed or endorsed by the publisher.

## Footnotes

^1^The terms people with disabilities (person first language) and disabled people (identity first language) are used interchangeably to reflect the current preferences of advocates in the disability rights field.
